# Human oropharynx as natural reservoir of *Streptobacillus hongkongensis*

**DOI:** 10.1038/srep24419

**Published:** 2016-04-14

**Authors:** Susanna K. P. Lau, Jasper F. W. Chan, Chi-Ching Tsang, Sau-Man Chan, Man-Ling Ho, Tak-Lun Que, Yu-Lung Lau, Patrick C. Y. Woo

**Affiliations:** 1 State Key Laboratory of Emerging Infectious Diseases, The University of Hong Kong, Hong Kong; 2 Department of Microbiology, The University of Hong Kong, Hong Kong; 3 Research Centre of Infection and Immunology, The University of Hong Kong, Hong Kong; 4 Carol Yu Centre for Infection, The University of Hong Kong, Hong Kong; 5 Collaborative Innovation Center for Diagnosis and Treatment of Infectious Diseases, Zhejiang University, Hangzhou, China; 6 Department of Paediatrics & Adolescent Medicine, The University of Hong Kong, Hong Kong; 7 Department of Clinical Pathology, Tuen Mun Hospital, Hong Kong

## Abstract

Recently, we reported the isolation of *Streptobacillus hongkongensis* sp. nov. from patients with quinsy or septic arthritis. In this study, we developed a PCR sequencing test after sulfamethoxazole/trimethoprim and nalidixic acid enrichment for detection of *S. hongkongensis.* During a three-month study period, among the throat swabs from 132 patients with acute pharyngitis and 264 controls, PCR and DNA sequencing confirmed that *S. hongkongensis* and *S. hongkongensis*-like bacteria were detected in 16 patients and 29 control samples, respectively. Among these 45 positive samples, five different sequence variants were detected. Phylogenetic analysis based on the 16S rRNA gene showed that sequence variant 1 was clustered with *S. hongkongensis* HKU33^T^/HKU34 with high bootstrap support; while the other four sequence variants formed another distinct cluster. When compared with the 16S rRNA gene of *S. hongkongensis* HKU33^T^, the five sequence variants possessed 97.5–100% sequence identities. Among sequence variants 2–5, their sequences showed ≥99.5% nucleotide identities to each other. Forty-two individuals (93.3%) only harbored one sequence variant. We showed that the human oropharynx is a reservoir of *S. hongkongensis*, but the bacterium is not associated with acute pharyngitis. Another undescribed novel *Streptobacillus* species is probably also residing in the human oropharynx.

*Streptobacillus* is one of the four genera within the family *Leptotrichiaceae.* It was first isolated from the blood of a patient who suffered from a rat bite in 1914^1^. Since the establishment of the genus *Streptobacillus* in 1925, this genus had contained only one single species, *Streptobacillus moniliformis*, for almost 90 years^2^. *S. moniliformis* is the causative agent of streptobacillary rat-bite fever^3^. It is also associated with amnionitis^4^, bacteremia^5^,^6^,^7^, brain abscess^8^, cutaneous and subcutaneous abscesses^9^,^10^, endocarditis^11^,^12^, female genital tract abscess^13^, palmoplantar pustulosis^14^, septic arthritis^15^,^16^,^17^, spinal epidural abscess^18^, splenic abscess^19^, spondylodiscitis with psoas abscess^20^, and synovitis^21^. Microbiologically, *S. moniliformis* is a Gram-negative facultative anaerobic bacillus that grows in chains. It is naturally harbored in the oral cavity^22^ and upper respiratory tract^23^,^24^ of rats.

In 2014, we reported the isolation of a novel *Streptobacillus* species, named *S. hongkongensis*, from the pus of a patient with quinsy and the elbow joint fluid of another patient with tophaceous gout and left elbow septic arthritis^25^. Both strains of *S. hongkongensis* were resistant to cotrimoxazole and nalidixic acid as determined by disk diffusion test (unpublished data). Since *S. moniliformis* colonizes the oral cavity of rats while *S. hongkongensis* could be isolated from peritonsillar abscess pus of human, we hypothesize that the oropharynx of human may be the natural reservoir of this bacterium or *S. hongkongensis* may be associated with acute pharyngitis. To test these hypotheses, we first developed an in-house molecular test for the detection of *S. hongkongensis* on throat swabs of patients with acute pharyngitis. Preliminary study showed that *S. hongkongensis* or *S. hongkongensis*-like sequences were detected in around 10% of 100 patient samples collected (unpublished data), indicating the presence of this bacterium in human oropharynx. In this molecular epidemiological study, in order to further test for the association of *S. hongkongensis* with acute pharyngitis, we used the in-house developed molecular method to examine for any significant difference between the detection rates of *S. hongkongensis* on throat swabs of patients with acute pharyngitis and healthy controls.

## Results

### Antimicrobial susceptibility

*S. hongkongensis* HKU33^T^ and HKU34 grew as white clumps of cells in brain-heart infusion (BHI) broth after 3 days of incubation under aerobic condition supplemented with 5% CO_2_ at 37 °C. Growth was observed for both strains at all drug concentrations tested for both sulfamethoxazole/trimethoprim and nalidixic acid. Sulfamethoxazole/trimethoprim at a concentration of 400/80 μg/ml and nalidixic acid at a concentration of 40 μg/ml were used for enrichment purpose.

### Primer specificity

**Figure 1:**
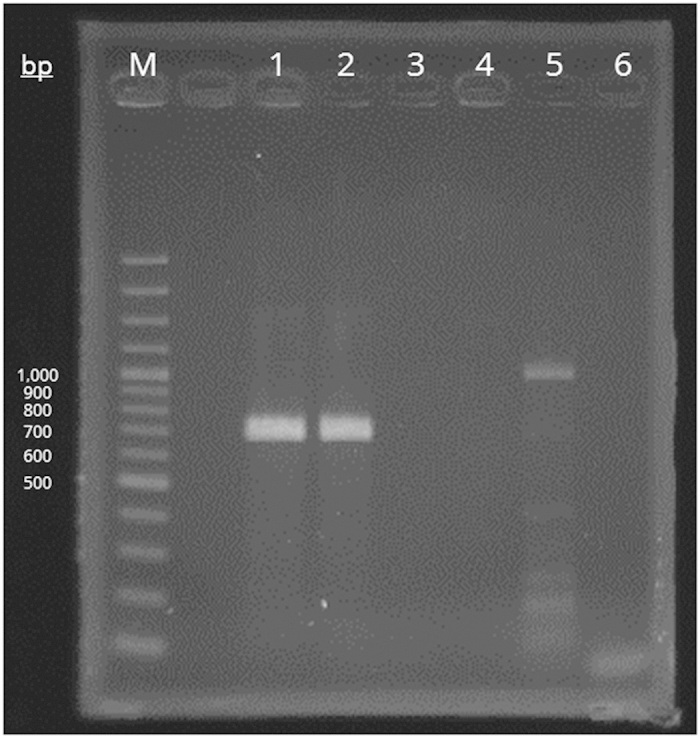
Photograph of ethidium bromide-stained agarose gel showing the PCR products of the partial 16S rRNA gene using the primer pair LPW21953/LPW21954. Lane M, DNA marker; lane 1, *Streptobacillus hongkongensis* HKU33^T^; lane 2, *S. hongkongensis* HKU34; lane 3, *S. moniliformis* CCUG 13453^T^; lane 4, *Sneathia sanguinegens* CCUG 41628^T^; lane 5, “Sneathia amnii” CCUG 52976; lane 6, negative control.

PCR of the partial 16S rRNA gene of *S. hongkongensis* strains HKU33^T^ and HKU34 using the primer pair LPW21593/LPW21594 yielded DNA fragments of about 700 bp with strong signal. For *S. moniliformis* CCUG 13453^T^, *Sneathia sanguinegens* CCUG 41628^T^, and “Sneathia amnii” CCUG 52976, no PCR product of expected size was detected (Fig. 1).

### Molecular detection of *S. hongkongensis* and *S. hongkongensis*-like bacteria

During the three-month period, 132 throat swab samples from 132 patients [male:female = 7:4, age (median, range) = 18.5 years, 0–92 years] with acute pharyngitis were sent to our clinical microbiology laboratory and 264 throat swab samples from 264 controls [male:female = 85:47, age (median, range) = 23 years, 0–95 years], including 132 healthy individuals and 132 out-patients not on antibiotics, without acute pharyngitis were included. Among the 132 patient and 264 control samples collected, PCR and DNA sequencing confirmed that *S. hongkongensis* and *S. hongkongensis*-like bacteria were detected in 16 patient and 29 control samples (Table 1).

### Sequence analysis and phylogenetic characterization

**Figure 2:**
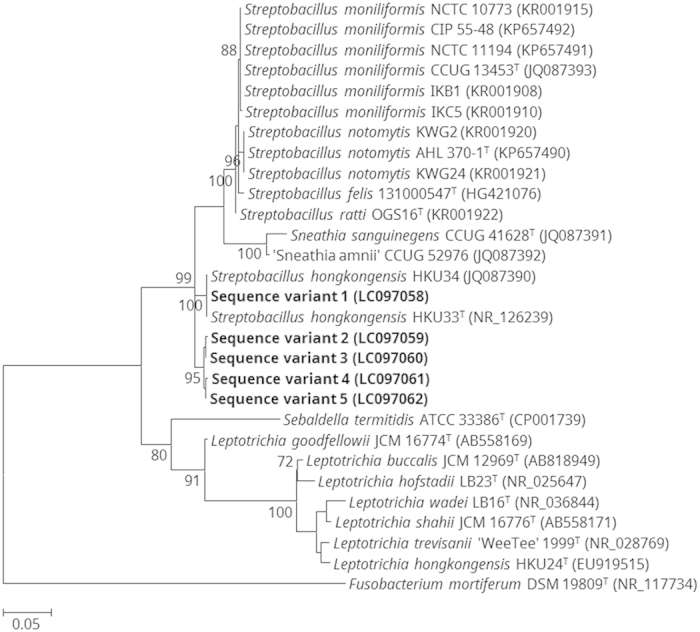
Phylogenetic tree showing the relationship of the five PCR-detected *Streptobacillus hongkongensis* sequence variants to other members of the family *Leptotrichiaceae*. The tree was inferred from 16S rRNA gene sequence data by the maximum likelihood method with the substitution model K2 (Kimura 2-parameter model) + G (gamma-distributed rate variation) + I (estimated proportion of invariable sites), and was rooted using *Fusobacterium mortiferum* DSM 19809^T^. The scale bar indicates the estimated number of substitutions per base. Numbers at nodes, expressed in percentage, indicate levels of bootstrap support calculated from 1,000 trees and bootstrap values lower than 70 are not shown. All accession numbers (in parentheses) are given as cited in the DDBJ/ENA/GenBank databases. The five *S. hongkongensis* sequence variants reported in this study are highlighted in bold type.

Among all of the 45 positive samples, five different sequence variants were detected. Phylogenetic analysis based on the 16S rRNA gene showed that sequence variant 1 was clustered with *S. hongkongensis* HKU33^T^ and HKU34 with high bootstrap support; while the other four sequence variants formed another distinct cluster (Fig. 2). When compared with the 16S rRNA gene of *S. hongkongensis* HKU33^T^, sequence variants 1, 2, 3, 4, and 5 possessed 100%, 97.5%, 97.7%, 97.5%, and 97.7% sequence identities, respectively. Among sequence variants 2, 3, 4, and 5, their sequences showed ≥99.5% nucleotide identities. Forty-two individuals (93.3%) only harbored one sequence variant (variants 1, 2 and 4). However, in one patient and one control sample, two sequence variants (variants 2 and 4) were detected while in another control sample all the five sequence variants were detected (variants 1–5).

## Discussion

In this study, we showed that human oropharynx is a reservoir of *S. hongkongensis*. Since numerous bacterial species reside in the human oropharynx, we developed a PCR test after sulfamethoxazole/trimethoprim and nalidixic acid enrichment, as the two *S. hongkongensis* strains we isolated previously, HKU33^T^ and HKU34, were resistant to these antibiotics. These antibiotics were also widely used as enrichment supplements for the detection of *Streptobacillus*-like bacteria previously^22^,^26^. Using this molecular method for detection, overall, *S. hongkongensis* (sequence variant 1), which possessed a 100% 16S rRNA gene sequence identity with those of the two previously isolated *S. hongkongensis* strains HKU33^T^ and HKU34^25^, was found in 2.0% of the throat swabs from all subjects tested. There was no significant difference between the detection rates for patients and controls, indicating that the bacterium is not associated with acute pharyngitis. All positive samples (sequence variant 1) were collected from children under the age of 14 years (*p* < 0.002 by Fisher’s exact test) (Table 1) without any sex predilection, suggesting children are the major population carrying the bacterium. On the other hand, it is notable that the two strains of *S. hongkongensis* which we previously isolated were recovered from the peritonsillar abscess pus of a 38-year-old man and the joint fluid of a 64-year-old man, respectively^25^, indicating that diseases caused by *S. hongkongensis* can occur in adults. Isolation and identification of more strains of *S. hongkongensis* from infective sites would reveal a more detail epidemiology and disease spectrum of this bacterium.

Another undescribed novel *Streptobacillus* species is likely also residing in the human oropharynx. In the present study, in addition to *S. hongkongensis* (sequence variant 1), four other 16S rRNA gene sequence variants (sequence variants 2–5), which possessed ≥99.5% nucleotide identities among themselves, 97.5–97.7% nucleotide identities to that of *S. hongkongensis*, and ≤95.9% nucleotide identities to that of the type strains of other *Streptobacillus* species, were detected. Since these four sequence variants form a unique cluster distinct from *S. hongkongensis* (sequence variant 1) as well as other known *Streptobacillus* species, it is likely that this represent another novel *Streptobacillus* species (Fig. 2). It is notable that the 16S rRNA gene sequences of *S. felis* and *S. notomylis*, the two recently described *Streptobacillus* species, possessed 99.1% nucleotide identity when compared with each other and ≥97.6% nucleotide identities to that of *S. moniliformis*^27^,^28^. However, subsequent DNA-DNA hybridization experiment and pairwise whole genome comparison showed that they are two separate species distinct from *S. moniliformis*^27^,^28^. Since the potentially novel *Streptobacillus* species (sequence variants 2–5) can be detected by the present molecular test, it should also be resistant to sulfamethoxazole/trimethoprim and nalidixic acid. Overall, this potentially novel *Streptobacillus* species was detected in 9.6% of the throat swabs from all subjects tested, suggesting that its prevalence in human oropharynx is higher than that of *S. hongkongensis*. Similar to *S. hongkongensis*, this potentially novel *Streptobacillus* species was also found primarily in subjects under the age of 14 (*p* < 0.001 by Fisher’s exact test), particularly in children below five years of age (*p* < 0.05 by Fisher’s exact test) (Table 1) with no sex predilection.

More novel *Streptobacillus* species could be discovered from other animals. The genus *Streptobacillus* had remained monotypic for almost a century^2^. In the last two decades, the use of 16S rRNA gene sequencing has led to the discovery of numerous novel bacteria^29^. In the past two years, four novel *Streptobacillus* species were described, including *S. hongkongensis*, *S. felis*, *S. notomylis*, and *S. ratti*. *S. felis* was isolated from the lung of a cat with acute suppurative to fibrinous, focally necrotizing bronchopneumonia with multifocal desquamation of type II pneumocytes and alveolar macrophages^28^; whereas *S. notomylis* from the heart of an Australian spinifex hopping mouse (*Notomys alexis*) with septicemia as well as the oral swabs of rats (*Rattus rattus*)^22^,^27^,^30^,^31^; and *S. ratti* from the oral swab of a rat^32^. In two recent animal microbiome projects, other potentially novel *Streptobacillus* species with ≥91.5% partial 16S rRNA gene sequence identities to known *Streptobacillus* species were also found in the oral cavities of a Cape ground squirrel (*Xerus inauris*) (accession number: HM590422, unpublished data) and dogs (*Canis familiaris*) (accession numbers: EU082091 and JN713542)^33^,^34^. In human, the two anatomical sites with the largest number of novel bacteria discovered in the recent two decades are the gastrointestinal tract and oral cavity^29^. For example, *Streptococcus sinensis*, a bacterium discovered in 2002^35^ and associated with infective endocarditis in patients from different parts of the world^35^,^36^,^37^,^38^, was finally also found to be part of human oral flora^39^. This indicates that there should be yet numerous novel bacterial species remained undescribed from the oral cavity. We speculate that the oral cavities and oropharynx of other animals might be the reservoirs of their own *Streptobacillus* species. We anticipate that more novel *Streptobacillus* species will be described and more potentially novel *Streptobacillus* species will be revealed by molecular epidemiology and microbiome studies in the next decade.

## Materials and Methods

### Antimicrobial susceptibility

The *in vitro* antimicrobial susceptibilities of *S. hongkongensis* against sulfamethoxazole/trimethoprim (range: 1.5625/0.3125 to 400/80 μg/ml) and nalidixic acid (range: 0.15625 to 40 μg/ml) were determined to test for the enrichment condition. Briefly, drug powders of sulfamethoxazole (Fluka, Switzerland), trimethoprim (Fluka), and nalidixic acid (Sigma-Aldrich, St. Louis, MO, USA) were dissolved in sterile dimethyl sulfoxide (Sigma-Aldrich) for the preparation of stock solutions. The stock solutions were then added into 5 ml of BHI broth (Oxoid, UK) to give a series of culture media supplemented with antimicrobial drugs at concentrations within the test range. Approximately 2.5 × 10^6 ^cfu of *S. hongkongensis* HKU33^T^ was then added into each of the test medium (final cell density = 5 × 10^5 ^cfu/ml) and the inoculated broths were incubated under an aerobic environment supplemented with 5% CO_2_ at 37 °C for 3 days. The presence of growth at each drug concentration was then recorded. The test was also performed using another *S. hongkongensis* reference strain, HKU34.

### Primer design for PCR detection

**Figure 3:**
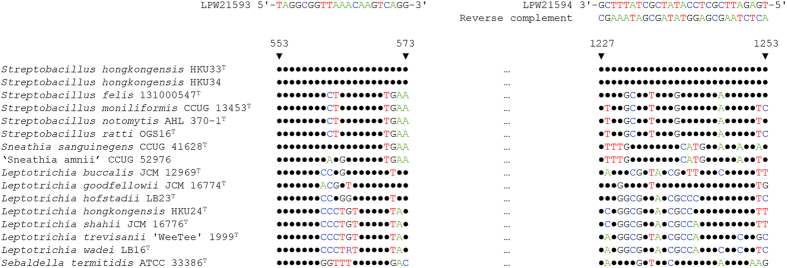
Sequence alignment of the PCR primer regions of the 16S rRNA genes of *Streptobacillus hongkongensis* and other members of the family *Leptotrichiaceae*. The nucleotide positions of the primer regions are shown with respect to the complete 16S rRNA gene of *S. hongkongensis* HKU33^T^ (DDBJ/ENA/GenBank accession number NR_126239).

A pair of primers [LPW21593 (5′-TAGGCGGTTAAACAAGTCAGG-3′) and LPW21594 (5′-TGAGATTCGCTCCATATCGCTATTTCG-3′)] (Invitrogen, Carlsbad, CA, USA) were designed by selecting the conserved regions of the 16S rRNA genes of *S. hongkongensis* HKU33^T^ and HKU34 (Fig. 3). The specificity of this primer pair was tested using the genomic DNA extracted from strains HKU33^T^ and HKU34, as well as the closely related reference strains *S. moniliformis* CCUG 13453^T^, *Sneathia sanguinegens* CCUG 41628^T^, and “Sneathia amnii” CCUG 52976, which were obtained from Culture Collection, University of Göteborg (CCUG), Sweden. Bacterial DNA was extracted using the alkaline lysis method. Briefly, cells from a single colony were resuspended in 200 μl of phosphate-buffered saline (PBS) (Oxoid), and 800 μl of NaOH (0.05 mol/L) (Sigma-Aldrich) was added into the bacterial suspensions. The cell suspension mixtures were then heated at 60 °C for 45 min. The lysed cell suspensions were then each neutralized by an addition of 240 μl of Tris-hydrochloric acid (pH 7.0) (Sigma-Aldrich). The neutralized suspensions were then centrifuged at 16,100 rcf for 5 min to remove the cell debris and the total bacterial DNA contained in the supernatant was collected and stored at −20 °C until use.

PCR amplification of the partial 16S rRNA gene was performed using the primer pair LPW21593/LPW21594. Briefly, each 25 μl-PCR reaction mixture contained UltraPure DNase/RNase-free distilled water (Invitrogen, Carlsbad, CA, USA), 1 μl of bacterial DNA, PCR buffer (10 mM of Tris-HCl [pH 8.3], 50 mM of KCl and 2 mM of MgCl_2_) (Applied Biosystem, Foster City, CA, USA), 1 mM of each primer, 200 μM of each dNTP (Roche Diagnostics, Switzerland), and 0.625 U of AmpliTaq Gold DNA polymerase (Applied Biosystem). The PCR reaction mixtures were first heated at 95 °C for 10 min, then heated in 45 cycles of 95 °C for 1 min, 52.5 °C for 1 min, and 72 °C for 1 min, and finally incubated at 72 °C for 10 min in the GeneAmp PCR System 9700 automated thermal cycler (Applied Biosystem). Five microliters of each amplified product were electrophoresed alongside with the DNA marker GeneRuler 100 bp Plus (Thermo Scientific, Waltham, MA, USA) in 1.5% (w/v) agarose gel (SeaKem LE Agarose) (Lonza, Switzerland) and after electrophoresis the agarose gel was stained with ethidium bromide (Sigma-Aldrich) for DNA band visualization.

### Ethics statement

This study has been approved by the Institutional Review Board (IRB) of the University of Hong Kong/Hospital Authority Hong Kong West Cluster and carried out according to the approved protocol. For the use of archived, left over patient throat swab specimens, the requirement of obtaining written informed consent from the patients was waived by the IRB; and no additional patient sample was collected. For the use of control samples, written informed consent was obtained from all the control subjects or their parents or legal guardians.

### Patients and controls

All throat swab samples from patients with acute pharyngitis sent to the clinical microbiology laboratory of Queen Mary Hospital, Hong Kong, during a three-month period (1 April 2015 to 30 June 2015) were included in the study. To determine if *S. hongkongensis* is associated with acute pharyngitis, controls without acute pharyngitis were recruited from healthy volunteers and out-patients. Subjects were excluded if they had immunocompromising conditions including primary immunodeficiency, systemic or inhalational steroid, other immunosuppressive drugs, chemotherapy, diabetes mellitus, chronic organ failure, thalassemia major with regular transfusions and/or iron overload, and hematopoietic stem cell or solid organ transplantation. Subjects who received antibiotics in the preceding 2 weeks prior to throat swabs collection were also excluded. All throat swabs were processed immediately after their arrival at the laboratory.

### Bacterial culture

Throat swabs were suspended in BHI broth supplemented with 400/80 μg/ml of sulfamethoxazole/trimethoprim and 40 μg/ml of nalidixic acid. The inoculated broths were then incubated under aerobic environment supplemented with 5% CO_2_ at 37 °C for 3–5 days.

### DNA extraction, PCR, cloning, and DNA sequencing

In order to extract total bacterial DNA from the broth cultures, cells in the broth suspensions were collected by centrifugation at 16,100 rcf for 10 min. Then, the cells were resuspended in 200 μl of phosphate-buffered saline (PBS), and DNA was extracted using the alkaline lysis method as described above. PCR of the partial 16S rRNA gene was performed using the primer pair LPW21593/LPW21594 as described above. DNA from *S. hongkonensis* HKU33^T^ and autoclaved distilled water were used as the positive and negative controls in each run of PCR, respectively. Preparation of PCR master mix, addition of DNA samples into the reaction tubes and post-PCR steps, including gel electrophoresis, purification of PCR products and DNA sequencing, were performed in separated rooms to avoid possible contamination. After agarose gel electrophoresis, the PCR products were purified using the QIAquick Gel Extraction Kit (QIAGEN) according to the manufacturer’s protocol. Both strands of the PCR products were sequenced twice with an ABI Prism 3700 DNA Analyzer (Applied Biosystems), using the respective PCR primers. The sequencing electropherograms obtained were viewed using Chromas Lite 2.1.1 (Technelysium, Australia).

When ambiguous peaks were observed in the sequencing electropherograms, the PCR products obtained were cloned into plasmids for another trial of sequencing, which was performed according to our previous publication^40^. Briefly, freshly-prepared gel-purified PCR products were cloned into pCRII-TOPO vector using the TOPO TA Cloning Kit (Invitrogen) according to the manufacturer’s protocol. The TA-ligated plasmids were then transformed into *Escherichia coli* DH5α (TaKaRa Bio, Japan) by electroporation. The electroporated cells were then grown on LB agar, Lennox (Difco, BD Diagnostic Systems, Sparks, MD, USA) with kanamycin (50 μg/ml) (Sigma-Aldrich) for the selection of positive transformants, as well as isopropyl β-D-1-thiogalactopyranoside (IPTG) (40 μg/ml) (Sigma-Aldrich) and 5-bromo-4-chloro-3-indolyl-beta-D-galactopyranoside (X-gal) (100 μg/ml) (Promega, Madison, WI, USA) for blue-white screening. White colonies were then selected and grown in LB broth, Lennox (Difco) with kanamycin (50 μg/ml) overnight and the plasmids in the bacterial cells were extracted using the QIAprep Spin Miniprep Kit (QIAGEN) according to the manufacturer’s protocol. The purified plasmids were then sequenced using the PCR primers directly. The sequencing electropherograms obtained were viewed using Chromas Lite 2.1.1.

### Comparative sequence identity analyses and phylogenetic analyses

The sequences obtained from the cloned PCR products were analyzed by pairwise alignment, with the optimal GLOBAL alignment parameters, using BioEdit 7.2.0^41^. The sequences of the PCR products were also compared with sequences of closely related species from the DDBJ/ENA/GenBank databases by multiple sequence alignment using MUSCLE 3.8^42^ and were then end-trimmed. Poorly aligned or divergent regions of the aligned, end-trimmed DNA sequences were removed using Gblocks 0.91b^43^ with relaxed parameters, and 653 nucleotide positions were included in the final sequence alignment dataset. Tests for substitution models and phylogenetic tree construction using the maximum likelihood method were performed using MEGA 6.06^44^.

### Nucleotide sequence accession numbers

The 16S rRNA gene sequences of the five *S. hongkongensis* sequence variants detected have been deposited in the DDBJ/ENA/GenBank databases with the accession numbers LC097058- LC097062.

## Additional Information

**How to cite this article**: Lau, S. K. P. *et al.* Human oropharynx as natural reservoir of *Streptobacillus hongkongensis*. *Sci. Rep.* **6**, 24419; doi: 10.1038/srep24419 (2016).

## Figures and Tables

**Table 1:** Prevalence of *Streptobacillus hongkongensis* and *S. hongkongensis*-like bacteria in diseased and healthy individuals in different age groups.

Age (year)	Number of positive samples/Total number of samples in each age group (Positive rate within each group)			
	*Streptobacillus hongkongensis*(Sequence variant 1)		*S. hongkongensis*-like bacteria(Sequence variants 2–5)	
	Patients	Controls	Patients	Controls
0–5	2/46 (4.3%)	4/62 (6.5%)	12/46 (26.1%)	17/62 (27.4%)
6–13	0/14 (0%)	2/54 (3.7%)	2/14 (14.3%)	7/54 (13.0%)
14+	0/72 (0%)	0/148 (0%)	0/72 (0%)	0/148 (0%)

## References

[b1] Schottmüller H. Zur atiologie und klinik der bisskrankheit (ratten-, katzen-, eichhornchen-bisskrankheit). Dermatol. Wochenschr. 58, 77 (1914).

[b2] Levaditi C., Nicolau S. & Poincloux P. Sur le rôle étiologique de *Streptobacillus moniliformis* (nov. spec.) dans l'érythème polymorphe aigu septicémique. C. R. Acad. Sci. 180, 1188–1190 (1925).

[b3] Blake F. G. The etiology of rat-bite fever. J. Exp. Med. 23, 39–60 (1916). 19867970 10.1084/jem.23.1.39 PMC2125348

[b4] Faro S., Walker C. & Pierson R. L. Amnionitis with intact amniotic membranes involving *Streptobacillus moniliformis*. Obstet. Gynecol. 55, 9S–11S (1980). 7360458 10.1097/00006250-198003001-00003

[b5] Torres L. *et al.* Bacteremia by *Streptobacillus moniliformis*: first case described in Spain. Eur. J. Clin. Microbiol. Infect. Dis. 22, 258–260 (2003). 12709841 10.1007/s10096-003-0891-9

[b6] Nei T. *et al.* *Streptobacillus moniliformis* bacteremia in a rheumatoid arthritis patient without a rat bite: a case report. BMC Res. Notes 8, 694 (2015). 26584844 10.1186/s13104-015-1642-6 PMC4653872

[b7] Okamori S. *et al.* A Japanese patient with a rare case of *Streptobacillus moniliformis* bacteremia. J Infect. Chemother. 21, 877–878 (2015). 26344724 10.1016/j.jiac.2015.08.003

[b8] Dijkmans B. A. C., Mattie H., Thomeer R. T. W. M., Vielvoye G. J. & Lampe A. S. Brain abscess due to *Streptobacillus moniliformis* and *Actinobacterium meyerii*. Infection 12, 262–264 (1984). 6490171 10.1007/BF01645956

[b9] Vasseur E., Joly P., Nouvellon M., Laplagne A. & Lauret P. Cutaneous abscess: a rare complication of *Streptobacillus moniliformis* infection. Br. J. Dermatol. 129, 95–96 (1993). 8369220 10.1111/j.1365-2133.1993.tb03323.x

[b10] Hagelskjaer L., Sørensen I. & Randers E. *Streptobacillus moniliformis* infection: 2 cases and a literature review. Scand. J. Infect. Dis. 30, 309–311 (1998). 9790145 10.1080/00365549850161016

[b11] Madhubashini M., George S. & Chandrasekaran S. *Streptobacillus moniliformis* endocarditis: case report and review of literature. Indian Heart J. 65, 442–446 (2013). 23993005 10.1016/j.ihj.2013.06.019 PMC3861209

[b12] Fenn D. W., Ramoutar A., Jacob G. & Bin Xiao H. An unusual tale of rat-bite fever endocarditis. BMJ Case Rep. 10.1136/bcr-2014-204989 (2014). PMC4244522 25414213

[b13] Pins M. R., Holden J. M., Yang J. M., Madoff S. & Ferraro M. J. Isolation of presumptive *Streptobacillus moniliformis* from abscesses associated with the female genital tract. Clin. Infect. Dis. 22, 471–476 (1996). 8852965 10.1093/clinids/22.3.471

[b14] Danion F., Bui E., Riegel P. & Goichot B. Streptobacillosis characterised by palmoplantar pustulosis. Lancet Infect. Dis. 13, 96 (2013). 23257235 10.1016/S1473-3099(12)70141-X

[b15] Wang T. & Wong S. *Streptobacillus moniliformis* septic arthritis: a clinical entity distinct from rat-bite fever? BMC Infect. Dis. 7, 56 (2007). 17561996 10.1186/1471-2334-7-56 PMC1903360

[b16] Flannery D. D., Akinboyo I., Ty J. M., Averill L. W. & Freedman A. Septic arthritis and concern for osteomyelitis in a child with rat bite fever. J. Clin. Microbiol. 51, 1987–1989 (2013). 23554193 10.1128/JCM.03139-12 PMC3716043

[b17] Budair B., Goswami K. & Dhukaram V. Septic arthritis secondary to rat bite fever: a challenging diagnostic course. BMJ Case Rep. 2014, 10.1136/bcr-2014-204086 (2014). PMC3987209 24695665

[b18] Addidle M., Pynn J., Grimwade K. & Giola M. Epidural abscess caused by *Streptobacillus moniliformis*. J. Clin. Microbiol. 50, 3122–3124 (2012). 22718932 10.1128/JCM.01004-12 PMC3421793

[b19] Chulay J. D. & Lankerani M. R. Splenic abscess: report of 10 cases and review of the literature. Am. J. Med. 61, 513–522 (1976). 973645 10.1016/0002-9343(76)90331-4

[b20] Dubois D. *et al.* *Streptobacillus moniliformis* as the causative agent in spondylodiscitis and psoas abscess after rooster scratches. J. Clin. Microbiol. 46, 2820–2821 (2008). 18562588 10.1128/JCM.00744-08 PMC2519479

[b21] Torres A. *et al.* Remitting seronegative symmetrical synovitis with pitting edema associated with subcutaneous *Streptobacillus moniliformis* abscess. J. Rheumatol. 28, 1696–1698 (2001). 11469482

[b22] Kimura M. *et al.* Detection of *Streptobacillus* spp. in feral rats by specific polymerase chain reaction. Microbiol. Immunol. 52, 9–15 (2008). 18352907 10.1111/j.1348-0421.2008.00005.x

[b23] Strangeways W. I. Rats as carriers of *Streptobacillus moniliformis*. J. Pathol. Bacteriol. 37, 45–51 (1933).

[b24] Paegle R. D., Tewari R. P., Bernhard W. N. & Peters E. Microbial flora of the larynx, trachea, and large intestine of the rat after long-term inhalation of 100 per cent oxygen. Anesthesiology 44, 287–290 (1976). 1259185 10.1097/00000542-197604000-00002

[b25] Woo P. C. Y. *et al.* *Streptobacillus hongkongensis* sp. nov., isolated from patients with quinsy and septic arthritis, and emended descriptions of the genus *Streptobacillus* and *Streptobacillus moniliformis*. Int. J. Syst. Evol. Microbiol. 64, 3034–3039 (2014). 24912824 10.1099/ijs.0.061242-0

[b26] Greenwood J. R. & Harvey S. M. Streptobacillus moniliformis In The Prokaryotes (eds Dworkin M., Falkow S., Rosenberg E., Schleifer K.-H., Stackebrandt E. ) 983–985 (Springer, 2006).

[b27] Eisenberg T. *et al. Streptobacillus notomytis* sp. nov. isolated from an Australian spinifex hopping mouse (*Notomys alexis*) THOMAS, 1922 and emended description of *Streptobacillus* Levaditi *et al.* 1925, Eisenberg *et al.* 2015 emend. *Int. J. Syst. Evol. Microbiol.* In Press, 10.1099/ijsem.0.000654 (2015). 26438009

[b28] Eisenberg T. *et al.* *Streptobacillus felis* sp. nov., isolated from a cat with pneumonia, and emended descriptions of the genus *Streptobacillus* and of *Streptobacillus moniliformis*. Int. J. Syst. Evol. Microbiol. 65, 2172–2178 (2015). 25858245 10.1099/ijs.0.000238

[b29] Woo P. C. Y., Lau S. K. P., Teng J. L. L., Tse H. & Yuen K. Y. Then and now: use of 16S rDNA gene sequencing for bacterial identification and discovery of novel bacteria in clinical microbiology laboratories. Clin. Microbiol. Infect. 14, 908–934 (2008). 18828852 10.1111/j.1469-0691.2008.02070.x

[b30] Hopkinson W. I. & Lloyd J. M. *Streptobacillus moniliformis* septicaemia in spinifex hopping mice (*Notomys alexis*). Aust. Vet. J. 57, 533–534 (1981). 7342940 10.1111/j.1751-0813.1981.tb05802.x

[b31] Eisenberg T. *et al.* Phenotypic and genotypic characteristics of members of the genus *Streptobacillus*. Plos One 10, e0134312 (2015). 26252790 10.1371/journal.pone.0134312 PMC4529157

[b32] Eisenberg T. *et al.* *Streptobacillus ratti* sp. nov. isolated from a black rat (*Rattus rattus*). Int. J. Syst. Evol. Microbiol. In Press, 10.1099/ijsem.0.000869 (2015). 26705259

[b33] Wouters E. G. H., Ho H. T. K., Lipman L. J. A. & Gaastra W. Dogs as vectors of *Streptobacillus moniliformis* infection? Vet. Microbiol. 128, 419–422 (2008). 18061376 10.1016/j.vetmic.2007.10.019

[b34] Dewhirst F. E. *et al.* The canine oral microbiome. Plos one 7, e36067 (2012). 22558330 10.1371/journal.pone.0036067 PMC3338629

[b35] Woo P. C. Y. *et al.* *Streptococcus sinensis* sp. nov., a novel species isolated from a patient with infective endocarditis. J. Clin. Microbiol. 40, 805–810 (2002). 11880397 10.1128/JCM.40.3.805-810.2002 PMC120286

[b36] Uçkay I. *et al.* *Streptococcus sinensis* endocarditis outside Hong Kong. Emerg. Infect. Dis. 13, 1250–1252 (2007). 17953105 10.3201/eid1308.080124 PMC2828085

[b37] Faibis F. *et al.* *Streptococcus sinensis*: an emerging agent of infective endocarditis. J. Med. Microbiol. 57, 528–531 (2008). 18349377 10.1099/jmm.0.47528-0

[b38] Seta V., Teicher E., Fortineau N., Ladouceur M. & Lambotte O. Endocardite infectieuse à *Streptococcus sinensis*. Med. Mal. Infect. 45, 56–57 (2015). 25481726 10.1016/j.medmal.2014.11.001

[b39] Woo P. C. Y. *et al.* The oral cavity as a natural reservoir for *Streptococcus sinensis*. Clin. Microbiol. Infect. 14, 1075–1079. 19330895 10.1111/j.1469-0691.2008.02083.x

[b40] Zhao Y. *et al.* Intra-genomic internal transcribed spacer region sequence heterogeneity and molecular diagnosis in clinical microbiology. Int. J. Mol. Sci. 16, 25067–25079 (2015). 26506340 10.3390/ijms161025067 PMC4632790

[b41] Hall T. A. BioEdit: a user-friendly biological sequence alignment editor and analysis program for Windows 95/98/NT. Nucleic Acids Symp. Ser. 41, 95–98 (1999).

[b42] Edgar R. C. MUSCLE: multiple sequence alignment with high accuracy and high throughput. Nucleic Acids Res. 32, 1792–1797 (2004). 15034147 10.1093/nar/gkh340 PMC390337

[b43] Castresana J. Selection of conserved blocks from multiple alignments for their use in phylogenetic analysis. Mol. Biol. Evol. 17, 540–552 (2000). 10742046 10.1093/oxfordjournals.molbev.a026334

[b44] Tamura K., Stecher G., Peterson D., Filipski A. & Kumar S. MEGA6: Molecular Evolutionary Genetics Analysis Version 6.0. Mol. Biol. Evol. 30, 2725–2729 (2013). 24132122 10.1093/molbev/mst197 PMC3840312

